# Dicyanogold Effects on Lymphokine Production

**DOI:** 10.1155/MBD.1999.301

**Published:** 1999

**Authors:** Katherine Tepperman, Pamela W. Roy, Brian F. Moloney, R. C. Elder

**Affiliations:** Departments of Biological Sciences and Chemistry and the Biomedical Chemistry Research Center University of Cincinnati Cincinnati OH 45221-0006 USA

## Abstract

Having identified dicyanogold(I) as a common metabolite of gold-based antiarthritis drugs, we are investigating the effects of the compound on the production of lymphokines. Handel, et al. 1 suggested that the transcription factor AP-1, critical to the production of a number of cytokines, might be the target for gold compounds because of a critical cysteine within its DNA binding region. Using Jurkat cells, an established cell line as a model for CD4^+^ lymphocytes, we have shown that dicyanogold inhibits the binding of AP-1 to DNA and inhibits the synthesis of IL-2 mRNA and protein. In a macrophage line, THP-1, which synthesizes IL-1β in response to mitogen, we have shown that dicyanogold inhibits the binding of a second transcription factor, CREB to DNA. Incubation of THP-1 cells with dicyanogold inhibits the production of IL-1β mRNA. These results suggest that the mechanism of action of gold drugs may be through their interaction with transcription factors necessary for the immune activation seen in Rheumatoid Arthritis.


### DICYANOGOLD EFFECTS ON LYMPHOKINE PRODUCTION

Katherine Tepperman*, Pamela W. Roy, Brian F. Moloney, and R.C. Elder

Departments of Biological Sciences and Chemistry and the Biomedical Chemistry Research Center, University of Cincinnati, Cincinnati OH 45221-0006

### ABSTRACT
Having identified dicyanogold(I) as a common metabolite of gold-based antiarthritis drugs, we are investigating the effects of the compound on the production of lymphokines. Handel, *et al.* [1] suggested that the transcription factor AP-1, critical to the production of a number of cytokines, might be the target for gold compounds because of a critical cysteine within its DNA binding region. Using Jurkat cells, an established cell line as a model for $CD4^+$ lymphocytes, we have shown that dicyanogold inhibits the binding of AP-1 to DNA and inhibits the synthesis of IL-2 mRNA and protein. In a macrophage line, THP-1, which synthesizes IL-1β in response to mitogen, we have shown that dicyanogold inhibits the binding of a second transcription factor, CREB to DNA. Incubation of THP-1 cells with dicyanogold inhibits the production of IL-1β mRNA. These results suggest that the mechanism of action of gold drugs may be through their interaction with transcription factors necessary for the immune activation seen in Rheumatoid Arthritis.

### INTRODUCTION
Although gold complexes have been used for over sixty years for the treatment of rheumatoid arthritis (RA), little is understood of their mechanism of action. For several years, we have been interested in determining the fate of different gold complexes after administration to patients. Using HPLC coupled to ICP-MS for gold-specific detection, we have shown that dicyanogold(I) is a common metabolite found in patients treated with myochrysine (gold(I) sodium thiomalate), solganol (gold(I) thiooglucose), or auranofin (triethylphosphinegold(I)tetraacetylthioglucose)[2]. It had been suggested that the formation of dicyanogold(I) might increase cellular uptake of gold[3]. Since we have now found this compound circulating in the blood of patients, we have begun studies to investigate the effect of dicyanogold(I) on cells which play a role in rheumatoid arthritis.

Rheumatoid arthritis is characterized by inflammation and ultimately by progressive destruction of the joints[4]. Abnormal accumulation of T lymphocytes occurs, particularly $CD4^+$ cells, along with large numbers of infiltrating macrophages. Macrophage activation is characterized by the production of a number of cytokines, for example Interleukin 1β (IL-1β) and Tumor Necrosis Factor (TNF). Actual destruction of the matrix of the joint results from the production of degradative enzymes such as collagenase and stromelysin by synovial fibroblasts. Recent treatments for RA have been focused on targeting the immune system[5]. For example, promising agents have included immunosuppressive drugs such as cyclosporine, monoclonal antibodies to $CD4^+$, and compounds such as tenidap, which has cytokine modulating activity. One aspect common to the regulation of many of these cytokines is the transcription factor Activator Protein-1 (AP-1). AP-1 binding sites are found in the promotor regions of a number of genes activated in RA, including IL-1β, IL-2, TNF, collagenase, and stromelysin[6, 7].

AP-1 includes a family of transcription factors which are dimers of Jun and Fos[7]. Dimerization occurs through the formation of leucine zippers, and dimers bind to AP-1 sequences on the DNA. Jun proteins include c-Jun, JunB and JunD, and are capable of forming homodimers or heterodimers with members of the Fos family, including c-Fos, Fra-1, Fra-2, *etc.* The complex is also known to interact with other transcription factors including CREB[8] and NFAT[9] to promote the synthesis of many of the cytokines. Because of the central role played by AP-1 in various stages of immune activation involved in RA, it was proposed[1] that AP-1 may be a good target for antiarthritis agents. Handel, *et al.* suggested that AP-1 might interact with gold complexes specifically because of the presence of the amino acid sequence KCR within the DNA binding region of Jun and some members of the Fos family[10-12]. The location of the cysteine (C) residue between a lysine (K) and an arginine (R) results in a lower $PK_a$ for the thiol because of the positive charge of the neighboring basic residues. Using an electrophoretic gel mobility shift assay (EMSA), Handel[1] demonstrated that myochrysine inhibited the binding of AP-1 to its consensus sequence. When the cysteine residue within the DNA binding region was changed to serine, myochrysine had no effect on the protein-DNA interaction. This indicated that the binding of the gold complex was through cysteine and that binding of gold to AP-1 could inhibit its ability to stimulate transcription.

We have carried out a number of experiments to test Handel's hypothesis using a gold complex known to circulate in patients, dicyanogold(I), and using immune system cells activated during Rheumatoid Arthritis, $CD4^+$ lymphocytes and macrophages. For each cell type, we have chosen a cytokine whose synthesis is dependent on AP-1. Jurkat cells were used as a model for $CD4^+$ lymphocytes because they can be stimulate by mitogens to produce IL-2. A monocyte line, THP-1, is stimulated by mitogens to produce IL-1β. In addition to AP-1, IL-1β requires another transcription factor CREB, which also contains a potentially reactive cysteine within its DNA binding region[13]. This report indicates that dicyanogold(I), a metabolite actually found in patients, inhibits the binding of both AP-1 and CREB to their respective DNA sequences. Incubation of cells with dicyanogold reduces the production of IL-2 by Jurkat cells and IL-1β, as predicted by Handel.

### MATERIALS AND METHODS

Cell Culture: Cell lines used were obtained from ATCC, Jurkat (ATCC #TIB-152), THP-1 (ATCC # TIB-202). Both cell types were grown in medium containing 89% RPMI 1640 with 10% fetal calf serum, both from Hyclone, with 1% antibiotic-antimicotic solution (Gibco Life sciences). Cells were grown in canted flasks (Corning) at 37°C in 5% $CO_2$-95% air atmosphere. Fresh medium was added every 2-3 days. Dicyanogold(I) (Aldrich) was generally used at 50 ppb. Mitogens, phorbol, 12-myristate, 13 acetate (PMA) and phytohemagluttinin (PHA) were obtained from Calbiochem. For stimulation of Jurkat cells, PMA was used at a final concentration of 50 μg/mL, PHA at 10 μg/mL. For THP-1 cells, PMA was used at a final concentration of 20 ng/mL.

Preparations of Nuclear Extracts: Cells were collected by centrifugation, THP-1 cells at 800 rpm for 5 minutes in IEC Centra-4B centrifuge, Jurkat cells at 3000 rpm in a GSA rotor using a Sorvall RC2-B. Subsequently, cells were rinsed in phosphate buffered saline, PBS (137 mM NaCl, 2.7 mM KCl, 4.3 mM $Na_2HPO_4$7 $H_20$, 1.5 mM $KH_2PO_4$) and centrifuged at 2,000 rpm for 10 minutes in a Sorvall RC2-B SS-34 rotor at 4°C. The pellet was resuspended in 2 ml of Buffer A (10 mM HEPES, 1.5 mM MgCl2, and 10 mM KCl) with 5 mM DTT (Sigma, MO) and 0.7 mM PMSF (Sigma, MO). The cells were incubated on ice for 10 minutes (cells swell), 25 μL of 5% nonidet P-40 was added, the cells were homogenized with a Type B (ball) Dounce homogenizer, poured into 15 ml polystrene centrifuge tube, and centrifuged for 10 minutes at 2000 rpm in Sorvall SS-34 rotor. The pellet was resuspended in 1 ml of Buffer B (5 mM HEPES, 26% glycerol, 1.5 mM $MgCl_2$, and 0.2 mM EDTA) with 5 mM DTT, 0.7 mM PMSF, and 0.3 M NaCl. The nuclear fraction was incubated on ice with rotation (100 rpm) for 45minutes at 4°C, centrifuged at 12,000 rpm for 20 minutes at 4° C. The supernatant, which represents the nuclear extract, was placed in liquid nitrogen, and stored at –70 Celsius. Protein amounts were quantified using the BioRad colorimetric assay, using coomassie blue G-250.

Electrophoretic Gel Mobility Shift Assay (EMSA): The AP-1 and CRE consensus oligonucleotides were purchased from Promega. A 59 base pair segment representing -2727 to -2785 of the IL-1β promotor sequence was synthesized by Operon. The oligonucleotides were end labeled with $^{32}P$ (NEN Life Sciences) using modifications of the Promega Protocol (Tech. Bulletin #519). For the AP-1 experiments, modifications of Promega Tech, Bulletin #110 were followed. Gels were polymerized overnight at 4°C and run at 4°C. For the CRE experiments, 10 μg protein was mixed with $^{32}P$ - oligonucleotide in a buffer containing 5mM HEPES, 3% glycerol, 0.2 mM EDTA, 0.3 mM $MgCl_2$, 10 mM spermidine (Aldrich), 5 mM DTT, 0.1% Nonidet P-40. All reagents unless otherwise indicated were obtained from Sigma. Incubations of probe and extract were carried out at room temperature for 15 minutes. When competitions were done, unlabeled competitor was added prior to the labeled probe. Samples were loaded onto polyacrylamide gels (0.5X TBE, 4.1% acrylamide, 0.1% bisacrylamide, 3.5% glycerol, 0.00075% ammonium persulfate 0.0012% TEMED; TBE: 90 mM Tris base, 90 mM boric acid, 10 mM EDTA, pH 8.0) and run at 180 V for one hour at room temperature. Gels were dries with a BioRad Gel dryer for one hour at 80°C, then placed on film (NEN Life Sciences) at -70°C for 18 hours.

RNA Extraction: Cells were collected from 100 mL of medium by centrifugation, then suspended in 10 mL of TRI-reagent® (Molecular Research Center, Cincinnati, OH) for 10 min at room temperature. One mL of 1-bromo-3-chloropropane was added and the mixture was further incubated at room temperature for 15 minutes. The mixture was centrifuged at 10,000 rpm in a SS-34 rotor in a Sorvall RC-2B, for 215 min at 4°C. The pellet was then washed with 70% ethanol, air dried, then resuspended in 200 µL FORMAzol® (Molecular Research Center, Cincinnati, OH), incubated at 55°C for 15 min, then stored at -20°C. RNA was analyzed for purity and quantified by UV absorption.

RNase Protection: IL-2 mRNA was quantified using a RiboQuant™ Multi-Probe RNase Protection assay system from PharMingen. Template set hCK-1 was used. All procedures were carried out according to the manual provided by the supplier[14]. After electrophoresis was complete, the gel was adsorbed to Whatman filter paper then dried for 55 min on a Savant Speed Gel SG2000 with heat and vacuum. The dried gel was placed on a phosphorimager plate and allowed to sit at room temperature for 24-48 hr. The image was scanned on a Molecular Dynamics Storm 860 scanner and quantified using Image Quant.

Northern Analysis: IL-1β mRNA was quantified by Northern blot. A 600 bp probe for IL-1β was cut from a YEpsec-hll beta plasmid (ATCC 67024) using Sma I and Bam H1 (New England BioLabs) sequentially. After digestion, the DNA was separated on a 1% low-melt agarose gel, run at 60V for 2.5 hours. The 600 bp fragment was cut out of the gel, then boiled in 3 µL of $dH_2O$ per mg of gel. The probe was labeled by the random hexanucleotide method[15, 16]. RNA was denatured at 65°C in denaturing buffer (250 µL formamide, 87.5 µL formaldehyde, 50 µL 10X MOPS; 10X MOPS: 0.2 M MOPS, 10 mM EDTA, 50 mM Sodium acetate) and was loaded onto 1% agarose gel with formaldehyde (6.7% formaldehyde, 1X MOPS, in 0.1%DEPC treated $dH_2O$) and run in 1X MOPS at 80$^V$ or 30 min then 52 V for 15 hours. RNA was transferred to a nylon membrane (GeneScreen, NEN Life Sciences) with 10X SSC (3 M NaCl, 0.3 M sodium citrate). The membrane was baked at 80°C for 1 hour and UV crosslinked. It was then incubated with the radioactive probe for 20 hours, rotating, at 65°C in 26 mL hybridization fluid (1% bovine serum albumin, 7% SDS, 0.55 M $NaPO_4$). The membrane was washed two times with a solution containing 1% BSA, 5% SDS, 40 mM $NaPO_4$, 1 mM EDTA for 10 min at 65°C, then washed two times with 0.05% BSA, 1% SDS, 40 mM $NaPO_4$ for 10 min at 65°C. The membrane was exposed to X-ray film at -70°C for 24 hours.

Enzyme Linked Immuno Sorbent Assay (ELISA): IL-2 was quantified using an immunoassay kit purchased from BioSource International and analyzed according to the manufacturer's protocols. The colorimetric reaction was monitored at 450 nm using a Vmax™ Kinetic Microplate Reader (Molecular Devices Corporation).

### RESULTS

Our initial experiments were based on the work of Handel, et at.[1], who suggested that the mechanism by which gold drugs may work is through interaction with transcription factors necessary for immune activation in RA. We began to test this hypothesis by using cells which are representative of those activated in RA. To model $CD4^+$ lymphocytes, we have used the Jurkat cell line[17] and for macrophages we have used the THP-1 cell line[18]. Each of these cell lines synthesizes a cytokine in response to mitogen stimulation, IL-2 in the Jurkat cells and IL-1β in the THP-1 cells. Activation of $CD4^+$ cells and macrophages is characteristic of RA. Thus, monitoring the effects of gold compounds on the production of these cytokines may provide information on the mechanism of action of gold.

Based on our previous work demonstrating that dicyanogold(I) is found circulating in patients independent of the type of gold drug administered[2], our initial experiments explored the effect of dicyanogold(I)on the interaction of transcription factors with their DNA recognition sequences. We first used an EMSA experiment to examine the effects of dicyanogold(I) added *in vitro* to purchased c-jun and a consensus AP-1 consensus oligonucleotide (Figure 1). In the absence of added protein, the DNA migrates toward the bottom of the gel. In the presence of a protein which binds the DNA, the DNA is retarded and thus found toward the top of the gel. At the lowest concentrations of dicyaongold(I), there is a dark band at the position of the c-jun + probe, indicating that there is a significant amount of protein in the reaction which can bind to the DNA sequence. Addition of dicyanogold(I) to c-jun at concentrations above 250 ppb significantly inhibits the binding of c-jun to its DNA sequence. Handel, et al.[1] used myochrysine in their experiments. These results indicate that dicyanogold(I) is also able to block c-jun binding to DNA.

The initial suggestion that AP-1 might react with gold was based on the presence of a cysteine within the DNA binding region of c-jun which was surrounded by a lysine on one side and an arginine on the other (KCR). It was suggested that the presence of the surrounding basic amino acids might make that cysteine more reactive to gold than others[1]. Our examination of the transcription factor requirements for expression of IL-1β by macrophages indicated that, in addition to AP-1 IL-1β expression requires CREB[19]. Since CREB has a cysteine within its DNA binding region also surrounded by basic amino acids (RCR) we tested whether CREB binding might also be affected by gold. For these experiments, we used a 59 bp oligonucleotide which is part of the IL-1β promoter and which includes a CRE sequence. The source of the transcription factor was a nuclear extract from THP-1 cells which had been stimulated by mitogen.

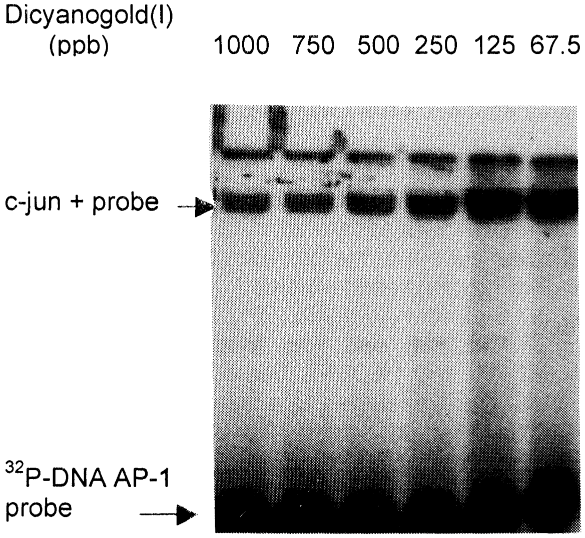

Figure 1. EMSA of Dicyanogold(I) Effect on Interaction of c-Jun with the AP-1-Consensus Sequence. c-jun was incubated with the indicated concentration of dicyanogold(I), then mixed with the AP-1 consensus oligonucleotide. The mixture was separated by electrophoresis as described in Materials and Methods. Positions of probe and c-jun + probe are indicated with arrows.

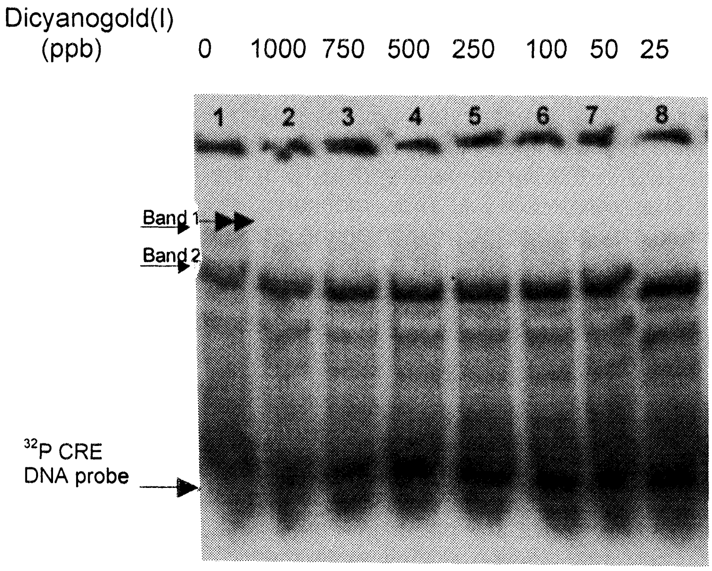

Figure 2. EMSA of Dicyanogold(I) Effect on CREB Interaction with DNA. THP-1 cells were activated with PMA, then nuclear extracts were prepared. Extracts were mixed *in vitro* with dicyanogold(I) at the indicated concentrations, then with the $^{32}$P-labeled 59 bp sequence from the IL-1β promoter. The gel was run as described in Materials and Methods. Bands 1 and 2 and CRE probe are indicated by arrows.

Figure 2 shows EMSA results. In these experiments, there are two shifted bands seen when the 59 bp fragment is reacted with nuclear extracts, labeled bands 1 and 2 in the figure. We observed bands at the same region of the gel when pure CREB protein was reacted with the consensus CRE oligonucleotide (data not shown), thus suggesting that both of these bands represent CREB binding. As seen with the c-jun experiment, dicyanogold(I) significantly inhibits the binding of one of the CREB bands. In both the c-jun and CREB EMSA experiments, some samples were incubated with potassium cyanide in concentrations comparable to those used for dicyanogold(I) (data not shown). Potassium cyanide has no effect on DNA binding in either case, indicating that the inhibition observed is due to the presence of gold. Thus, dicyanogold(I), a compound which circulates in patients, blocks the interaction of both c-jun and CREB with their respective DNA sequences.

Having established that both c-jun and CREB interact with dicyanogold *in vitro*, we asked whether exposure of whole cells to dicyanogold would alter the amount of DNA binding in nuclear extracts from either type of cell. To do these experiments, Jurkat cells or THP-1 cells were exposed to 50 ppb dicyanogold(I) for varying times. To activate the cells, cells were exposed to the appropriate mitogens, Jurkat cells for three hours, THP-1 cells for seven. Nuclear extracts were then prepared and used for EMSA experiments with the appropriate oligonucleotide. Figures 3 and 4 illustrate the results of these experiments.

Nuclear extracts from Jurkat cells show a significant amount of binding to the AP-1 consensus DNA (Figure 3). When these cells were exposed to mitogen, there was a significant increase in the amount of material at the position of the gel indicating DNA binding. This suggests that mitogen stimulation significantly increased the amount of AP-1 present in the nucleus and capable of binding to DNA. When cells were incubated with dicyanogold(I), there was some reduction of DNA binding relative to the control sample. In these experiments, the amount of protein loaded on the gels was equivalent in all cases. Nuclear extracts from cells stimulated by mitogen in the presence of dicyanogold(I) have much less material in the protein/DNA band on the gel compared to those from cells stimulated in the absence of gold. This indicates than dicyanogold(I) incubation reduces the amount of AP-1 protein in the nuclear extract which is capable of binding to DNA.

Nuclear extracts from THP-1 cells stimulated with mitogen show similar effects to those observed with Jurkat cells. Figure 4 shows the EMSA results. The results are similar to those seen with AP-1.

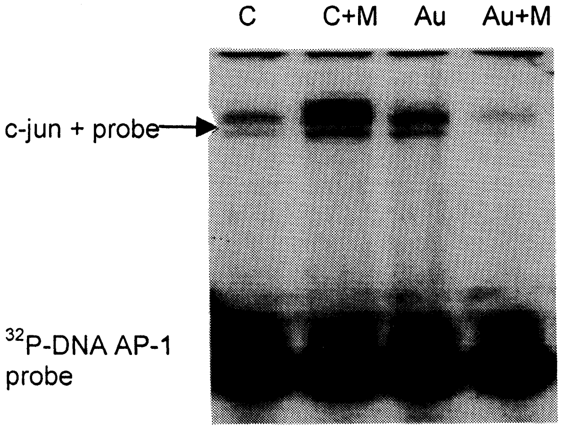

Figure 3. EMSA of Nuclear Extracts from Jurkat Cells exposed to dicyanogold(I). Jurkat cells were incubated in 50 ppb dicyanogold(I) for 24 hours, then exposed to mitogens for three hours. Nuclear extracts were prepared, then mixed with $^{32}$P consensus oligonucleotide and separated on acrylamide gels. Arrows indicate the relative positions of free probe and probe with c-jun bound.

Nuclear extracts from cells incubated with mitogen show significantly more radioactivity in Band 1. Incubation of the cells in dicyanogold(I) significantly inhibits the observed increase in intensity of the band. Interestingly, in the case of nuclear extracts from cells treated with dicyanogold(I) Band 1 is much more significantly affected by gold than Band 2, a similar result to that seen when extracts were exposed to gold *in vitro*. The reduction in Band 1 material after dicyanogold(I) treatment was also observed in cells incubated for up to one week prior to mitogen treatment (data not shown). Thus, cells continue to respond to dicyanogold(I) treatment by decreasing the amount of CREB available in the nucleus to bind DNA for a significant period of time.

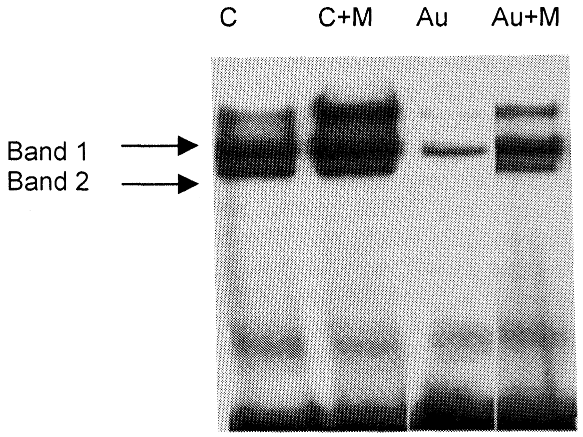

Figure 4. EMSA of Nuclear Extracts of THP-1 Cells Incubated with Dicyanogold(I). Experiment was carried out as in Figure 3. Probe used was CRE consensus sequence. Bands 1 and 2 are indicated by arrows. Individual lanes were cut out and reordered to make this figure easily compared to the previous figure.

For the observed effects on DNA binding of transcription factors to be physiologically significant, it would be predicted that incubation of cells with dicyanogold(I) would lead to a decrease in the production of mRNA for the cytokines which depend on the specific transcription factor for their synthesis. We have looked at this question in both Jurkat cells and THP-1 cells, monitoring the effect of dicyanogold(I) incubation on synthesis of a cytokine mRNA. For Jurkat cells, we used a ribonuclease protection assay. Cells were incubated in dicyanogold(I) for 24 hours, then incubated with mitogens for three hours. The Ribonuclease Protection assay was carried out using a series of probes for human cytokines. For these experiments, human glyceraldehyde 3-phosphate dehydrogenase (GAPDH) was used as an internal control. Densities in IL-2 mRNA and GAPDH bands were compared. The results of one such experiment are shown in Figure 5.

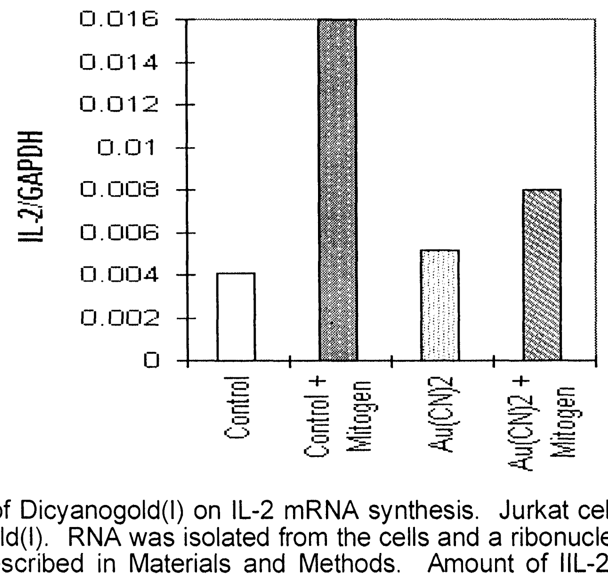

Figure 5. Effect of Dicyanogold(I) on IL-2 mRNA synthesis. Jurkat cells were incubated for 24 hrs in 50 ppb dicyanogold(I). RNA was isolated from the cells and a ribonuclease protection experiment was carried out as described in Materials and Methods. Amount of IIL-2 mRNA was compared to the amount of GAPDH mRNA as an internal control.

In cells not exposed to either mitogen or dicyanogold(I) (Control), very little IL-2 mRNA is detectable. When cells are exposed to mitogen for three hours, there is a significant stimulation of IL-2 mRNA production. Cells incubated in dicyaongold(I) with no mitogen have essentially the same amount of IL-2 mRNA as the control cells. Incubation of the cells with dicyanogold(I) followed by mitogen leads to a significant reduction in the amount of IL-2 mRNA produced. In four experiments carried out, the level of IL-2 mRNA present was approximately 50% of that seen in cells with mitogen stimulation alone.

Similar experiments were carried out using the THP-1 cells to monitor the effects of dicyanogold(I) on IL-1β mRNA production. These experiments were conducted by Northern analysis, since the amount of the mRNA of interest was significantly higher. For these experiments, cells were exposed to dicyanogold(I) for varying periods of time, then incubated with mitogen for seven hours. The seven hour time point was chosen because mRNA expression is maximal in these cells at that time (data not shown). After mitogen treatment, RNA was extracted and the Northern analysis was carried out as described in Materials and Methods. In each lane, the amount of total RNA was equalized in the loading of the gels, so varying intensity of the bands represents varying amounts of IL-1β mRNA as a fraction of the total RNA. The results of a typical experiment are shown in Figure 6. Cells which were not exposed to either mitogen or dicyanogold(I) make no detectable IL-1β mRNA. Addition of dicyanogold(I) has no effect on mRNA production (lanes 3,5,7,9). Incubation of cells with mitogen (lane 2) resulted in significant detectable IL-1β mRNA (lane 2). Cells exposed to dicyanogold(I) for 24 hrs to 1 month show significantly less IL-1β mRNA than is seen in the cells treated with mitogen alone. Quantitation of the band densities in Figure 6 allows for a determination of the relative degree of IL-1β induction in gold treated cells at varying times. In the RNAs from cells incubated with dicyanogold(I) for 24 hours, the level of IL-1β mRNA was approximately 40% of that seen in cells treated with mitogen alone. At 3 days, the value was 85%, at 1 week 47%, and at 1 month 29%. These results show that dicyanogold(I) treatment significantly reduces the amount of IL-1β mRNA made in response to mitogen.

| | | | | | | | | | | |
|---|---|---|---|---|---|---|---|---|---|---|
| Mitogen | - | + | - | + | - | + | - | + | - | + |
| 50 ppb Au | - | - | 24h | 24h | 3d | 3d | 1w | 1w | 1m | 1m |

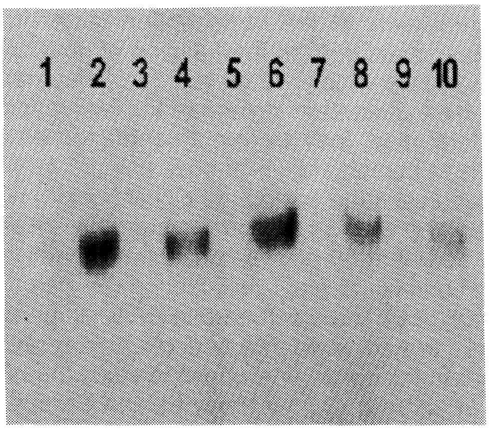

Figure 6. Effect of Dicyanogold(I) Incubation on Expression of IL-1β mRNA. Cells were incubated in 50 ppb dicyanogold(I) for the times indicated. Mitogen treated cells were exposed to PHA for seven hours, then the RNA was extracted and analyzed by Northern Blot, as described. Band densities were analyzed with ScionImage program

In addition to the observed effects on mRNA in the two cellular models, we have also investigated the effect of dicyanogold(I) on the production of IL-2 by the Jurkat cells. Again, we incubated the cells for varying periods of time in 50 ppb dicyanogold(I). We then stimulated the cells with mitogen and monitored the medium for the release of IL-2 protein using the Elisa Assay described in Methods. Figure 7 shows the averaged results from two experiments. For these two experiments, there is a significant decrease in IL-2 production by mitogen-stimulated cells which were also exposed to dicyanogold(I). Thus, dicyanogold(I) can be shown to inhibit the production of one of the cytokines whose synthesis is dependent upon AP-1 transcription factor.

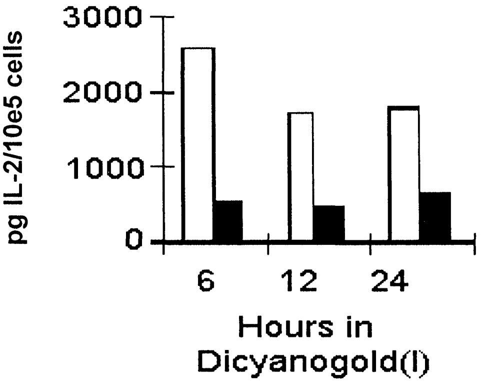

Figure 7. Effect of Dicyanogold(I) on Production of IL-2 by Jurkat Cells. Cells were incubated in dicyanogold(I) for varying times, then exposed to mitogen. Il-2 release into the medium was monitored by an Elisa assay. Open bars represent IL-2 production by cells exposed to mitogen but no dicyanogold(I). Solid bars represent IL-2 production by cells exposed to both mitogen and dicyanogold(I).

**Discussion**

These experiments were begun in an effort to test the hypothesis proposed by Handel, et al.[1] They have suggested that AP-1 is a target for gold drugs because of the presence of a particularly reactive cysteine within is DNA binding region. They had shown that myochrysine inhibited the binding of AP-1 to its target oligonucleotide and that removal of the target cysteine by mutagenesis removes the gold sensitivity of the transcription factor in its DNA binding. They further suggested that AP-1 inhibition could explain the function of gold drugs, since AP-1 is central to the activation of a number of cytokines which are elevated in Rheumatoid Arthritis. Inhibition of AP-1 binding to DNA then would be expected to decrease the activation of the cytokines. While their studies provided valuable information, they were not conducted on cells which were relevant to RA. They also used myochrysine, a compound which does not circulate in patients[2].

For our experiments, we have used the compound dicyanogold(I) which we have shown to be a common metabolite for the three know gold compounds currently in use[2]. For the experiments reported here, the compound was added at 50 ppb, a concentration well within the range which has been observed in patient fluids, and one which has little effect on the growth of the cultured cells. Cell culture models representative of the types of cells activated in RA were used. We used Jurkat cells as a model for $CD4^+$ cells. Jurkat cells are activated to produce IL-2 in response to mitogen, mimicking the response of activated $CD4^+$ cells in the body. We chose to study the effect of dicyanogold(I) on il-2 both because of its central role in activation of lymphocytes and because it has a promoter region which contains several binding sites for AP-1[9]. Since all sites must be occupied to provide maximal activation of transcription, it seemed as though blocking AP-1 binding to DNA with gold would be likely to affect IL-1 mRNA and subsequent protein production. Our first experiments paralleled those of Handel, et al.[1], and we showed that dicyanogold(I), like myochrysine, can reduce the binding of AP-1 to its target oligonucleotide. This result was seen, not only in nuclear extracts exposed to dicyanogold(I) *in vitro* (Figure 1), but was also seen when whole cells were incubated with dicyanogold(I) and nuclear extracts were subsequently reacted with DNA (Figure 3). Dicyanogold(I) not only interfered with AP-1 binding to DNA but it also inhibited the mitogen-induced stimulation of IL-2 mRNA (Figure 5) and IL-2 protein (Figure 7), as predicted by Handel.

In addition to the work with Jurkat cells, we also conducted experiments with THP-1 cells, a monocyte line which can be activated to macrophages. Mitogen-induction results in the activation of IL-1β, another cytokine observed at high levels in RA. IL-1β mRNA production required not only AP-1 but also CREB, the cyclic AMP response element binding protein[13]. CREB contains a cysteine within its DNA binding region which is chemically similar to the cysteine within the DNA binding region of AP-

1. The CREB cysteine is surrounded on both sides by arginine, and thus likely to be similarly reactive to the KCR cysteine in AP-1. We therefore tested the effect on dicyanogold(I) on the interaction of CREB with its oligonucleotide target. Using either a purchased CRE consensus sequence or an oligonucleotide found within the IL-1β promoter, we showed that CREB binding to DNA is inhibited by dicyanogold(I). Again, this is true whether the CREB is exposed to the gold compound *in vitro* (Figure 2) or after incubation of whole cells with the compound (Figure 4). The level of the gold which affects the *in vitro* transcription factor/DNA binding is similar for the two transcription factors. Significant inhibition is seen above 200 ppb dicyanogold(I). Since we have shown that both of these cell types concentrate gold (data not shown), the concentrations at which the inhibition is observed *in vitro* are observed in whole cells exposed to 50 ppb dicyanogold(I).

In addition to inhibition of CREB binding to DNA, we observed that exposure of cells to dicyanogold(I) inhibits the mitogen-induced production of IL-1β mRNA by about 50% after 24 hours of treatment in the compound. This level of inhibition is seen in cells exposed to dicyanogold(I) up to 1 month. Interestingly, dicyanogold inhibits IL-2 production by Jurkat cells to approximately the same degree (about 50%). While this level clearly does not represent a complete inhibition of either mRNA, it may suggest that gold complexes modulate the activation of the immune response rather than suppress it entirely.

These experiments extend the work of Handel, et al.[1] and provide support for their hypothesis that gold drugs may work through their interaction with transcription factors. Developing drugs which interact with transcription factors may represent a new approach to drug design.

**REFERENCES**

1. Handel, M. L.; Watts, C.K.W.; deFazio, A.; Day, R.O.; Sutherland, R.L. *Proc. Nat. Acad. Sci.* **92**: 4497-4504 (1995).
2. Elder, R.C.; Zhao, Z.; Zhang, Y.; Dorsey, J.G.; Hess, E.V.; Tepperman, K. *J. Rheumatol.* **20**: 268-272 (1993).
3. Graham, G.G.; Havisto, T.M.; Jones, H.M.; Champion, G.D. *Biochem. Pharmacol.* **33**: 311-323 (1984).
4. Gay, S.; Gay, R.E.; Koopman, W. *Annal. Rheum. Dis.* **52**: S39-S47 (1993).
5. Schiff, M. *Am. J. Med.* **102 (suppl. 1A)**: 11S-15S (1997).
6. Angel, P.; Imagawa, M,; Chiu, R.; Stein, B.; Imbra, R.; Rahmsdorf, H.; Jonat, C.; Herrlich, P.; Karin, M. *Cell* **49**; 729-739 (1987).
7. Foletta, V.C.; Segal, D.H.; Cohen, D.R. *J. Leuk. Biol.***63**: 139-152 (1998).
8. ref for interaction with CREB
9. Rooney, J.W.; Sun, Y.; Glimcher, L.H.; Hoey, T. *Molecular and Cellular Biology* **15**:6299-6310 (1995).
10. Hattori, K.; Angel, P.; LeBeau, M.M.; Karin, M. *Proc. Nat. Acad, Sci.* **85**: 9148-9152 (1988).
11. Ryder, K.; Lanahan, A.; Perez-Albuerne, E.; Nathans, D. *Proc. Nat. Acad. Sci.* **86**: 1500-1503 (1989).
12. Kawakami, Z.; Kitabayshi, I.; Matsuoka, T., Gachelin, G.; Yokoyama, K. *Nucl. Acids Res.* **20**: 914 (1992).
13. Benbrook, D.; Jones, N. *Nucl. Acids Res.* **22**: 1463-1469 (1995).
14. Pharmingen *Riboquant™ instruction Manual 3rd Edition* (1997).
15. Feinberg, A.; Vogelstein, B. *Anal. Biochem.* **132**: 6-13 (1983).
16. Feinberg, A.; Vogelstein, B. *Anal. Biochem.* **137**: 266-267 (1984).
17. Weiss A.;Wiskocil R.L.; Stobo J.D. *Journal of Immunology.* **133**: 123-8 (1984)
18. Tsuchiya S.; Yamabe M.; Yamaguchi Y.; Kobayashi Y.; Konno T.; Tada K. *Int.J.Cancer.* **26**:171-6 (1980).
19. Lorenza, J.; Furdon, P.; Taylor, J.; Vergese, M.; Chandra, G.; Kost, T.; Haneline, S.; Roner, L.; Gray, J. *J. Immunol.* **155**: 8360844 (1995).

**Received: October 3, 1998 -**
**Accepted in final form: February 19 1999**

